# Health worker migration and universal health care in Sub-Saharan Africa

**Published:** 2011-12-15

**Authors:** Isidore Sieleunou

**Affiliations:** 1 Health policy and financing specialist, Ministry of Public Health, Yaoundé, Cameroon

**Keywords:** Health workers, workforce, brain drain, Africa

## Abstract

There is a more and more emerging consensus claiming universal access to health care in order to achieve the desired Millennium Development Goals related to health in Africa. Unfortunately, the debate of the universal coverage has focussed so far mainly on financial affordability, while it is also a human resource matter. Many countries in sub-Saharan Africa are experiencing severe shortages of skilled health care workers. There are several causes, the importance of which varies by country, but one of the most significant factors is brain drain. In those countries, scarcity of doctors increases the distance between a doctor and patients, and bridging that increased distance implies costs, both time and money. Adequate number of qualified health personnel is then vital to increase coverage and improve the quality of care. In as much as access to health services is also determined by access to qualified health workers, any reflection on the universal health coverage has to also consider the inequities in qualified health personnel distribution throughout the world.

## To the editors of PAMJ

Less than one-third of the way into the deadline for the Millennium Development Goals, there is an emerging consensus claiming universal access to health care in order to achieve desired results related to health in Africa. It is very clear that universal coverage is vital. Having been born either poor and/or in an isolated village should not mean one is deprived of appropriate health care. If poverty is a strong determinant of health inequity, universal coverage is associated with better health status of the population, including the disfavoured [1].

The current context of globalisation benefits some people more than others, and prompts questions about inequity as we become aware that the problems of the poor populations in the south are partly due to the policies of the north. This is also true in the health sector.

Whatever name it goes by, be it selective immigration policy in France (“politique d'immigration choisie”), qualified workers immigration policy in Canada, immigration by lottery procedure in the United States of America (USA), brain circulation by some people, or any other name used elsewhere, these policies all have a common basis: encouraging the movement of qualified human resources from poor to rich countries. Found amongst these qualified migrants are many well-trained health personnel searching for greener pastures.

In sub-Saharan Africa, the problem of insufficient well-trained medical personnel is rampant and is associated with a risk of maternal mortality 30-50-fold higher than that of the rich countries; life expectancy at birth in sub-Saharan Africa is 54 years, 26 years lower than in high income countries today [2], and only three years higher than in the United States a century ago [3]. This flux of the brightest intellectuals represents a huge transfer of wealth from the poor to the rich countries. The resulting financial gains for the rich countries, as well as the financial losses for poor countries attributable to brain drain are quite appalling [4,5]. Agwu rightly qualified this as an “annoying subvention” [6] and McArthur as “reverse foreign aid” [7]. Mass emigration of physicians from less-developed countries results in even greater pressure on those who remain in these countries.

Besides these direct financial costs – which are accompanied by some positive inputs in terms of remittance and improvement of collaborative binds with more-developed countries – there are also indirect costs. For instance, the scarcity of doctors worsened by the voracious appetite of healthier economic nations for developing country health workers, increases the distance between a doctor and patients, and bridging that increased distance implies costs, both time and money. Meanwhile, it is widely recognized that an adequate number of qualified health personnel is vital to increase coverage and improve the quality of care. Both financial and human resources (amongst others) are required for universal health coverage to be attained in developing countries. However, human resources are dwindling due to depletion in their numbers following predatory recruiting policies (amongst other reasons) put in place by rich countries.

As globalisation increases interdependence between countries, the argument for global taxation approaches is becoming more and more pertinent [1]. A step in the right direction has been taken with the 1997 Durban Declaration and more recently in 2009 with the WHO executive committee who looked at the possibility of adopting a practical global code in response to the movement of health personnel from developing countries [8]. However, these initiatives do not sufficiently tackle the consequences of the selective brain drain of qualified personnel from developing countries.

In as much as access to health services is mainly determined by access to qualified health workers, any reflection on the global health gaps has to also consider the inequities in their distribution throughout the world. Hence, some authors have proposed developing adequate mechanisms for compensation or reimbursement of consequent losses [9]. But, obviously, the less-developed countries have also to learn to channel the potential of their own people by providing the fitting incentives for them to stay and excel.

The Universal Coverage debate must encompass all aspects. We have focussed so far mainly on financial affordability in the universal coverage. If we are not cautious to think about the universal coverage to the three areas; namely financial, service availability and appropriate adequate health personnel, we would always find gabs.

In doing so, we need henceforth to think “outside the box”. A new discourse is necessary in order to determine the direction in which our society should progress. We cannot stop (nor should we) the desire of individuals to look for a more enjoyable quality of life for themselves and their relatives. While waiting for the outcome of great debates and their implementation that is unlikely of in the near future, a first step could simply be the commitment to shared responsibilities for shared benefits that provides a window of opportunities to advocate that rich countries support access to universal health coverage for the world′s most impoverished citizens.
